# Effects of a skin-massaging device on the *ex-vivo* expression of human dermis proteins and *in-vivo* facial wrinkles

**DOI:** 10.1371/journal.pone.0172624

**Published:** 2017-03-01

**Authors:** Elisa Caberlotto, Laetitia Ruiz, Zane Miller, Mickael Poletti, Lauri Tadlock

**Affiliations:** 1 L’Oréal Research and Innovation, Chevilly Larue, France; 2 L’Oréal Research and Innovation, Aulnay sous Bois, France; 3 L’Oréal Research and Innovation, Redmond, WA, United States of America; University of Alabama at Birmingham, UNITED STATES

## Abstract

Mechanical and geometrical cues influence cell behaviour. At the tissue level, almost all organs exhibit immediate mechanical responsiveness, in particular by increasing their stiffness in direct proportion to an applied mechanical stress. It was recently shown in cultured-cell models, in particular with fibroblasts, that the frequency of the applied stress is a fundamental stimulating parameter. However, the influence of the stimulus frequency at the tissue level has remained elusive. Using a device to deliver an oscillating torque that generates cyclic strain at different frequencies, we studied the effect(s) of mild skin massage in an *ex vivo* model and *in vivo*. Skin explants were maintained *ex vivo* for 10 days and massaged twice daily for one minute at various frequencies within the range of 65–85 Hz. Biopsies were analysed at D0, D5 and D10 and processed for immuno-histological staining specific to various dermal proteins. As compared to untreated skin explants, the massaging procedure clearly led to higher rates of expression, in particular for decorin, fibrillin, tropoelastin, and procollagen-1. The mechanical stimulus thus evoked an anti-aging response. Strikingly, the expression was found to depend on the stimulus frequency with maximum expression at 75Hz. We then tested whether this mechanical stimulus had an anti-aging effect *in vivo*. Twenty Caucasian women (aged 65-75y) applied a commercial anti-aging cream to the face and neck, followed by daily treatments using the anti-aging massage device for 8 weeks. A control group of twenty-two women, with similar ages to the first group, applied the cream alone. At W0, W4 and W8, a blinded evaluator assessed the global facial wrinkles, skin texture, lip area, cheek wrinkles, neck sagging and neck texture using a clinical grading scale. We found that combining the massaging device with a skin anti-aging formulation amplified the beneficial effects of the cream.

## Introduction

Mechanical and geometrical cues influence cell behaviour and play a central role in embryogenesis, tissue physiology, and in a wide variety of diseases [1].

Mechanobiology is a branch of biology that focuses on the mechanisms by which physical forces induce changes in cells or tissues, through mechano-transduction, that may contribute to their development, influence physiologies, or induce disease. Understanding these mechanisms, i.e. the specific links between a physical stimulus and a molecular response, is a complex task that still remains unfinished. Many biophysically active molecules (actin, myosin, keratins, collagen, elastin, etc.) could potentially be altered by a mechanical stress, leading to extended and/or contracted forms [2–7]. Crane and colleagues [8] showed, through a clinical study and histology, that massage has an anti-inflammatory effect at the muscle level (with an effect on nucleoporin 88), and accelerates the synthesis of mitochondria.

At a tissue level, almost all organs exhibit immediate mechanical responsiveness and increase their stiffness in direct proportion to applied mechanical stress [9]. With regard to skin, previous work showed that mechanical stimulation generates a biological response in the tissue, particularly from fibroblasts [10–13].

The practice of massage and its impacts upon body heat, peripheral blood flow, autonomic nervous system function, and muscular strength has also been investigated [14–18]. However, there have been only a few studies on the effects of massage on skin structure or function [19–21] and none on the effects of massage on skin aging.

Skin aging is a complex, multifaceted biological process that is influenced by both internal and external factors. For example it is influenced by changes in hormonal status (steroids/menopause, glucocorticoids) and the local immune system [22,23], as well as by ultraviolet (UV) exposure and hypothalamic-pituitary-adrenal axis modulation [24–26]. The skin aging process globally reduces the production of extracellular matrix (ECM) proteins with the most pronounced effect being the degradation of the elastic fiber network, particularly at the dermal-epidermal junction (DEJ). The protein tropoelastin is the fundamental building block of all elastin and is required for all elastogenesis [27]. Microfibrils, of which fibrillin-1 is the major component, are structures present in the extracellular matrix which are thought to anchor elastic fiber formation. Elastic fibers are particularly important to the overall elasticity of the skin [28]. On the other hand, the DEJ zone of human skin represents an architectural link and a functional continuum between the epidermis and the dermis [29]. Fragility of DEJ is associated with aging and may lead to reduced nutrient transfer between the dermal and epidermal layers. Basement membranes are supramolecular composites of two independent but physically connected networks whose quantitatively major components are laminins and isoforms of collagen IV. The laminin and collagen IV containing networks are linked to each other to form functional basement membranes. It was recently shown that the core protein of perlecan and its heparan sulfate chains are parts of the laminin and collagen IV containing networks respectively [30]. Collagen VII, the anchoring fibril collagen which extends from the lower portion of the dermal-epidermal basement membrane to the underlying upper papillary dermis [31], and collagen IV, a major component of the basement membrane, are both involved in anchoring fibril structure and are produced by both keratinocytes and fibroblasts. They interact together and with other proteins to provide a molecular framework that confers mechanical strength to the skin. Synthesis of both these molecules decreases with age [32]. Procollagen 1 (precursor of collagen I), which binds fibronectin [33], and decorin are other important actors on the dermal fibril network. Decorin is a small, leucine-rich proteoglycan that binds collagen I, collagen III, fibronectin, and TGF-β [34,35]. It was shown that decorin KO mice have fragile skin with markedly reduced tensile strength, irregular collagen fibers with an abnormal diameter, and a less packed collagen network [34].

These considerations led us to envisage the development of a hand-operated massaging device that could be routinely used by consumers, alone or coupled to a cosmetic preparation. For the mechanical action, we focused on an oscillating torque at a fixed frequency and a small angular amplitude of oscillation (a few degrees), thereby delivering a short duration massaging action to a given skin surface (face, neck, legs, etc.). It was recently shown by different teams [10,36–38], that the frequency of mechanical stress is a fundamental stimulating parameter, in particular for cutaneous fibroblasts.

Two successive phases of development of this equipment were carried out. A first phase, carried out *ex vivo*, was aimed at identifying the influences of frequency and amplitude on the expression of certain dermal and/or DEJ structural proteins involved in the biology of skin aging. Once optimal ranges for frequency and amplitude were determined, these were adopted in a preindustrial device, referred to here as the “massaging device”. In a second phase, this device was used daily *in vivo*, in an evaluator-blinded, controlled clinical study, following the pre-application of a commercial anti-aging cream. Twenty healthy Caucasian women aged 65y-75y were randomly assigned to use the massaging device, combined with an anti-aging cream, over a two month-period. Another sub-group of twenty-two healthy Caucasian women of similar ages applied the cream alone. Clinical grading of global facial wrinkles, skin texture, lip area, cheek wrinkles, neck sagging and neck texture was performed by a blinded evaluator at weeks 4 and 8 compared to baseline (W0). The results of these two successive phases, *ex vivo* and *in vivo*, are the objects of the present paper.

## Materials and methods

### Ethics statement

For the *ex vivo* study, normal human skin remnants were obtained from abdominal surgical residues after written informed consent from the donors, in full respect with the Declaration of Helsinki and the article L.1243-4 of the French Public Health Code. The latter does not require any prior authorization by an ethics committee for sampling and using surgical wastes. These remnants, obtained from 8 Caucasian French women (free from any systemic medication), aged 32-68y, produced 121 skin explants (5 cm x 5 cm) that were used at different times, according to their availabilities. Normal human skin remnants were encoded with a number (code number) by the surgeon. The remnants were supplied with the code number and only the sampling region, sex and age of the donor were given to our laboratory (the donor could not be identified). Each experiment was repeated on two donors to confirm the results.

For the *in vivo* study, all subjects were informed about the objective of the study and signed an informed consent. The in vivo study was reviewed and approved by Western Institutional Review Board.

### Ex vivo

#### Protocol

**Fig 1:**
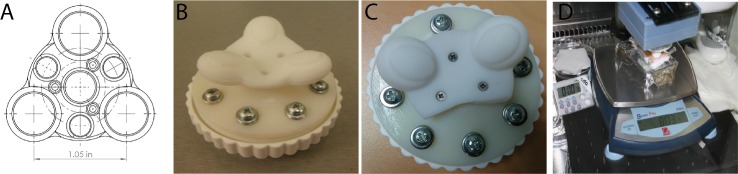
Treatment system. (A) A technical drawing of the massage head, showing the distance between the contact points of 2,5cm. (B): The massage head with 3 contact points used in *in vivo* study. (C) The massage head used in the ex vivo experiments, with only two massage points spaced 2,5cm apart. (D) The configuration set up for the ex vivo experiments: The Smart PROfile handle is connected to a micrometer staging system to control the position of the device on the skin. The pressure applied was measured with a scale and standardized at 80g. The entire set up was mounted on an anti-vibration table.

Skin was disinfected before moving to the operating theater. After surgery, the skin was put in a transport medium (DMEM + penicillin/streptomycin + amphotericin B). Upon receipt, the skin was washed in PBS and the epidermis surface was wiped with paper towels. The explants were then deposited onto non-woven MEFRA gauzes that were placed in 10 cm diameter Petri dishes, filled with 15 ml of a maintenance medium (DMEM + 10% fetal bovine serum, Sigma F4135, replaced daily, supplemented with penicillin/streptomycin) and incubated at 37°C, 5% CO2. These conditions allowed the explants to be maintained and massaged for 10 days in a preserved biological state (i.e. absence of necrosis). Before applying the massaging device, the explants were lifted from the gauze, put on a cork plate placed on an anti-vibration table, and tensioned by needles to ensure the flattest positioning (Fig 1D). Explants were massaged for 1 minute, twice daily (morning and evening), for 10 days at variable frequencies between 65 and 85 Hz with either 3° or 7° of angular oscillation. Control (unmassaged) explants were submitted to the same procedure. A constant and controlled pressure applied to the explants during massages was calibrated at 80g. After each massage treatment, explants were replaced into conditions of incubation. No differences were observed in skin biopsies from older vs younger donors in all explants during the 10 days of survival status.

#### Laboratory equipment

All experiments were performed using commercially available, production line versions of the Clarisonic^TM^ Smart PROfile oscillating brush handles. The system utilizes a flex-pivot, resonant, oscillatory motor with a natural frequency of approximately 175 Hz. The natural frequency of these motors is dependent on the ratio of the spring rate to the inertia of the moving parts of the system. The following equation governs the natural frequency

fn=kj2π

where *f*_*n*_ is the natural frequency, *k* is the spring rate, and *j* is the inertia of the system about the axis of rotation. From this equation, the resonant frequency of the system can be changed either by modifying the stiffness of the springs or modifying the inertia of the system. Increasing the spring rate will increase the resonant frequency while increasing the inertia by adding or redistributing mass will reduce it and vice versa. In these experiments, the resonant frequency of the system was reduced to the desired operating range by the addition of mass in the end effector. The Smart PROfile includes a radio frequency identification (RFID) antenna that allows it to query end effectors that include an RFID tag and adjust motor operating parameters, such as frequency and duty cycle, as indicated. In this way, the desired operating frequency and duty cycle (to control angular amplitude) was communicated to the handle for each experiment. The microcontroller in the handle would then modify the drive frequency and duty cycle of the motor to operate at the desired frequency and amplitude of the end effector.

The end effector used was a tri-lobular, Shore 30A silicone attachment with three contact points spaced 2.5cm apart, center-to-center (Fig 1A–1C).

This dimension was selected to maximize the shear-wave propagation through the ex-vivo sample, by avoiding half-wave cancellation. For these experiments, in order to maximize the number of samples available from a single donor, one of the three contact points was removed (Fig 1C).

#### Determination of frequency and amplitude of the massaging device

To determine the exact frequency and amplitude of stimulation applied to the skin samples as the wave propagates through the full depth of the tissue, an ultrafast ultrasound device (Aixplorer, Supersonic Imagine, Aix-en-Provence, France) using an 8 MHz central frequency probe (256 elements, 0.2 μm pitch, SL15-4, Supersonic Imagine, Aix-en-Provence, France) in research mode [39] was used *in vivo* on forearm on two healthy volunteers (of 26 and 42yo). Two frequencies were tested: 65 and 85Hz with 7deg of angular oscillatory amplitude.

#### Biopsies

At D0, D5 and D10, three 8 mm punch biopsies were taken from sites between the tips of the end effector (approx. 1.3 cm from each contact point) on the massaged explants and on their corresponding un-massaged explants, as controls. Biopsies were cut into two pieces, one piece being embedded in a Tissue Freezing Medium (Leica, ref: 14020108926), frozen in liquid nitrogen and stored at -80°C. The second piece was formalin-fixed and paraffin-embedded under conventional procedures.

#### Histological procedures

**Hematoxylin-Eosin-Saffron (HES) staining:** The first biopsy portion was rinsed and fixed by a formaldehyde solution, dehydrated in multiple baths of increasing concentrations of ethanol, and then embedded in paraffin. Five 5μm sections of each sample were obtained using a microtome and stored at room temperature prior to HES staining under conventional protocols. Post staining, the sections were rinsed, dehydrated, and mounted in CV Ultra medium (Leica, Wetzlar,‎ Germany).

**Immunofluorescent labeling:** Five 5μm sections of the frozen/embedded skin samples, separated by at least 50μm, were obtained using a cryo-microtome and stored at -80°C prior to immunolabeling of certain skin structural proteins or glycoproteins. The sections were fixed for 10 min at -20°C with an acetone/methanol mix (80%/20%) and then dried. After saturation in PBS-T-milk 5% for 30 minutes at room temperature, the sections were incubated for 1 hour at room temperature with the primary antibody (Table 1) prepared in PBS-T-milk 1%. After several washes, the binding sites were revealed for 1 hour at room temperature using the appropriate secondary antibody (Alexa Fluor 488; Table 1) and cell nuclei were stained with a propidium iodide solution (Sigma P4170). The sections were washed in a PBS-T solution and mounted in Fluorescent Mounting Medium (Dako). A negative control was performed without primary antibody.

**Table 1:** Summary of the various antibodies/protein label used in the ex vivo histological study.

Primary antibody	Supplier	Host	Reference	Dilution	Secondary antibody
**Anti-collagen IV**	Dako	mouse	M0785	1/50	GAM Alexa 488 A11001
**Anti-collagen VII**	Chemicon	mouse	MAB1345	1/50	GAM Alexa 488 A11001
**Anti-fibronectin**	Sigma	rabbit	F3648	1/100	GAR Alexa 488 A11008
**Anti-laminin 5**	Chemicon	mouse	MAB1947	1/100	GAM Alexa 488 A11001
**Anti-perlecan**	Invitrogen	mouse	13–4400	1/100	GAM Alexa 488 A11001
**Anti-procollagen I**	Chemicon	rat	MAB1912	1/100	GARat Alexa 488A11006
**Anti-tropoelastin**	Elastin products company	rabbit	PR398	1/100	GAR Alexa 488 A11008
**Anti-decorin**	R&D Systems	mouse	MAB143	1/100	GAM Alexa 488 A11001

Sections were observed using a NIKON E400 microscope (objective lens x40). The resulting images were captured with a NIKON DS-Ri1 and processed with NIS-Elements 4.13.04 software (Nikon Instruments, Inc., Melville, NY). Three different fields of each skin section were analysed, using ImageJ software (National Institutes of Health, Bethesda, Maryland) that quantified the fluorescence intensity for each biomarker and its corresponding labeled surface. To simplify the representation, only one donor per study is showed in the figures.

Comparison of the values of the fluorescent intensity of each marker to those from the corresponding control was performed with an analysis of variance (ANOVA test) with a p<0.05 threshold with Bonferroni adjustment.

### In vivo

#### Equipment

The devices used in the clinical study in Redmond (WA, USA) were standard production model Clarisonic^TM^ Smart PROfile handles, identical to the devices used in the *ex vivo* studies described above. The end effector (i.e. the massage head) used in the clinical studies was an updated version of the design used in the ex-vivo studies, redesigned to allow for better conformity to the contours of the face. The end effector was made of the same Shore 30A silicone and treated with a Parylene surface coating to reduce friction on the skin. The mass of the end effector was modified to bring the natural frequency of the system to 75 Hz. Frequency and duty cycle for the motor was controlled by an RFID tag attached to each end effector in the study, which set operating frequency to 75 Hz and angular amplitude to approximately 3°peak-to-peak unloaded.

#### Subjects

Forty-two Caucasian American women, aged 65y-75y, skin type I-III with moderate to severe facial wrinkles and facial sagging were enrolled. Only those who met the following criteria were included: (1) have no medically diagnosed chronic skin condition; (2) have not undergone any facial rejuvenation procedures within the year (e.g. intense pulsed light (IPL) laser, filler, etc.) or have had a botox treatment 6 months prior to their baseline appointment; (3) have no immunosuppression/immune deficiency disorders or uncontrolled disease such as diabetes, hypertension, hyperthyroidism, or hypothyroidism; (4) not be hypersensitive or have known allergies to skin care products. Study participants were randomized into two sub-groups with comparable age distributions; one group (Group 1) used the study cream applied manually, the other group (Group 2) used the study cream with the sonic skin-massaging device. The relative age distribution of these two groups is provided in Table 2. The purpose of this evaluator-blinded, controlled clinical study was to assess the added benefit of the sonic skin-massaging device over use of the study cream alone.

**Table 2:** Composition and age-distribution of the studied subjects.

Sub-Groups	Regimen	n	Age (mean ± SD)
**1**	Cream alone	22	69.3±3.3
**2**	Cream + stimulator	20	68±2.5

#### Protocol

All 42 subjects were asked to apply a blinded, commercially available Montanov™ based anti-aging cream, daily over a two-month period. Subjects in Group 1 were asked to apply the study cream twice daily, per their usual habits, to their whole face, neck and neck line. Subjects from Group 2 were each given a sonic skin-massaging device (Fig 1B) to apply the study cream to their whole face, neck and neckline. Subjects in Group 2 were instructed to use the prototype massage applicator to apply the study facial cream for 2 minutes and 15 second (30 seconds on the forehead, 1 minute around the mouth and check area, and 45 seconds along the jawline and neck) each time they used the device (both in the morning and before bed).

#### Clinical assessments

Clinical grading of several attributes was performed by a blinded evaluator using the modified Griffiths’ 10-point scale (0 = absence, 9 = severe) at D0 (prior to any application and or massage), D30 and D60. The attributes assessed were global facial wrinkles, skin texture, lip area wrinkles, cheek wrinkles, neck sagging, and neck texture. A questionnaire was completed by subjects from both groups immediately after the first application (S2 and S3 Tables), at Week 4 and Week 8 (S4 and S5 Tables), aimed at collecting their self-perceptions and opinions on various aspects of the skin massaging device (tolerance, easiness of use, comfort, etc.).

#### Statistics

Clinical scores for each parameter were averaged and expressed as mean ± S.D. (Standard Deviation). Clinical scores at 4 and 8 weeks (for both treatment groups) were compared to their respective baseline scores using Friedman’s Multiple Pairwise Comparison (p = 0.05) to determine if each treatment group had a statistically significant improvement from baseline for each clinical parameter. The two treatment groups were then compared to each other by calculating the change (Δ) from baseline scores at 4 and 8 weeks (D0 scores minus 4 or 8 week scores, reported as Mean Change from Baseline ±S.D. for each parameter and time point). For each clinical parameter and time point, the two groups were compared using Dunn’s Multiple Pairwise Comparison (p = 0.05) to determine if there were any statistically significant differences in improvement between the two treatment groups.

## Results

### Determination of frequency and amplitude of the massaging device

**Fig 2:**
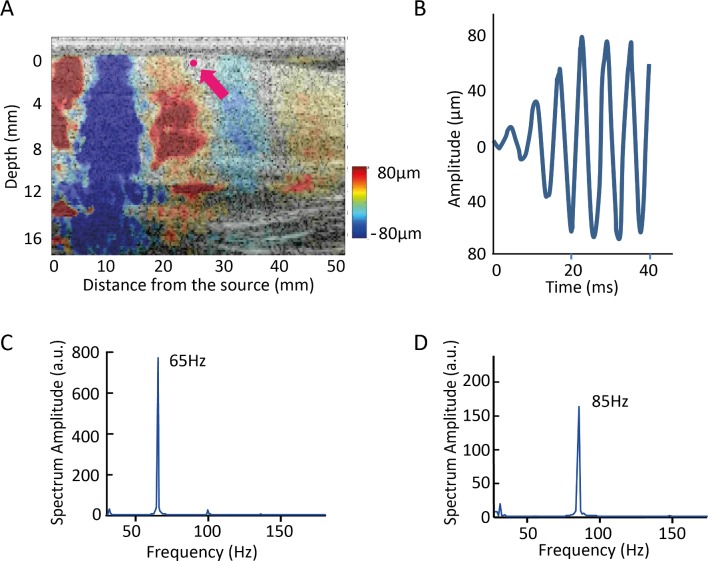
Determination of frequency and amplitude of the massaging device *in vivo*. The frequency and amplitude was verified *in vivo* in the forearm with ultrafast ultrasound imaging. (A) Spatiotemporal spreading of vibrations generated by the device at 65 Hz in the forearm of healthy volunteer of 26yo. The frequency and the amplitude of the displacement imposed to the skin were measured at a point 25 mm far from the source and 500μm depth on the tissue (the exact position is marked with an arrow). (B) The amplitude of the vibration measured the point marked in pink in *A* is about 150μm peak to peak. (C) The spectrum of the displacement field (averaged calculated in the full dermis and expressed in Arbitrary Units) shows that the fundamental frequency is 65 ± 3 Hz. (D) The spectrum of the displacement field (averaged calculated in the full dermis and expressed in Arbitrary Units) shows that the fundamental frequency is 85 ± 3 Hz.

The vibration wave generated by the device set at 65Hz and 85Hz were analysed *in vivo* (forearm) with ultrafast ultrasound imaging. From the spatiotemporal spreading recorded (Fig 2A) we were able to extract the spectrum of vibration (Fig 2C and 2D) and determine the frequency and the amplitude (Fig 2B) of the vibration propagating into the skin. The frequency measured in the skin is the same imposed by the massaging device stimulating the surface of the forearm (the mechanical wave propagates into the skin without a change in frequency). The amplitude of the vibration damps and depends on the position analysed. For example in Fig 2, a point 25mm away from the source and 500μm depth in the tissue experienced a vibratory wave with about 150μm of amplitude.

### Ex vivo

#### Histological analysis

**Fig 3:**
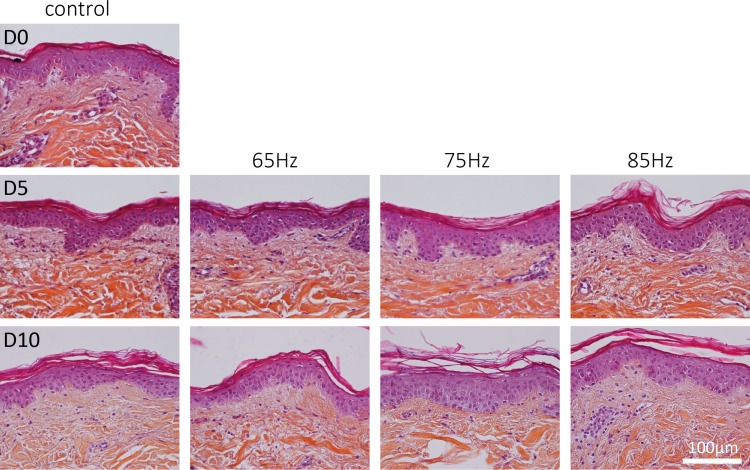
Haematoxylin-eosin stain-Safran (HES). Histological pictures of explants treated at various stress frequencies at a 3° peak-to-peak amplitude. All pictures shown are collected from one 33 year old donor. A second 32 year old donor was analysed with similar results (data not shown).

Observing the histological slides of all explants after HES staining (control and massaged), allowed the assessment of changes occurring over a 10 day-period (Fig 3). As compared to D0, the histological slides for to the control at D10 show that collagen structures are slightly damaged after 10 days of culture. On the other hand, this is not observed for the slides treated with the massaging device (in particular at 75Hz and 85Hz), in which the collagen structures appear clearer. Of note, at D10, the papillary dermis looks modified and apparently denser with the 75Hz treatment. A similar effect is observed with the 85Hz treatment. This indicates that the structures of the skin explants are affected by the massage treatment.

#### Expression of proteins

**Fig 4:**
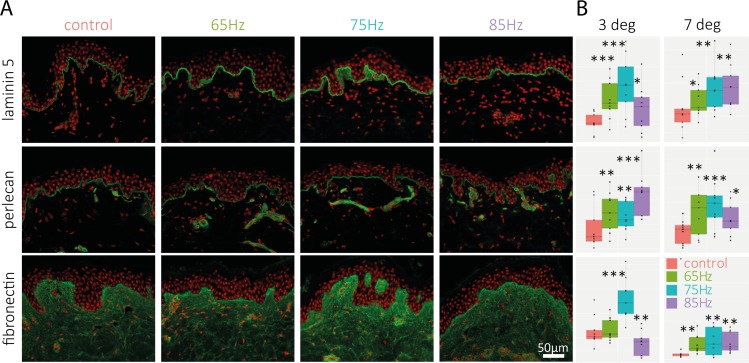
Immuno-labeling of DEJ and Dermal proteins for 65-85Hz treatment. (A) Immuno-labeling of laminin-5, perlecan and fibronectin (green labeling) at D5 for three tested frequencies with a 3° peak-to-peak angular amplitude; cell nuclei are stained with a propidium iodide solution (red labeling). Data shown are collected from one 33 year old donor, after 5 days of massaging. A second 32 year old donor was analyzed with similar results (data not shown). (B)Box Plot representation of the fluorescence intensity (arbitrary scale) of the measured markers for each condition tested at D5. Data shown are collected from two donors: 3 degree data were collected from a 33year old donor, and 7deg data were collected from a 32year old donor. The stars indicate the statistical significance of the labeling quantification for each condition compared with untreated skin (***: p<0.001, **: p<0.01 *: p<0.05).

Certain proteins in the dermis and/or DEJ were found to be expressed at higher levels when stimulated by the massaging device under our experimental conditions. Fig 4A illustrates the different fluorescent intensities of some proteins. Fig 4B quantifies the influences of variable massaging frequencies upon the 3 proteins markers analysed. In short, the expression of some proteins at Day 5 and Day 10 increased significantly as compared to their respective controls. Fig 4 shows that the amount of laminin 5 is clearly increased after the treatment at 75Hz compared to the control and the other frequencies tested, suggesting a reinforcement of the DEJ. A similar effect is observed for perlecan labeling, but the effect is less pronounced. In the dermis, fibronectin labeling signal is strongly increased by the 75Hz treatment, in particular in the papillary dermis, which is consistent with the structural changes observed in histological analysis present in Fig 3. The statistical analysis of the fluorescence quantification confirms that the expression of these markers appear higher when combining a 75Hz frequency with 3° or 7°angular amplitude. Other explored frequencies from previous experiments (less than 60 Hz and greater than 90Hz) had much less impact on the expression of these proteins as compared to their respective controls (data not shown). This led to the focus on the 60-90Hz range described in this study. No significant differences were observed between 3° and 7° angular amplitudes.

**Fig 5:**
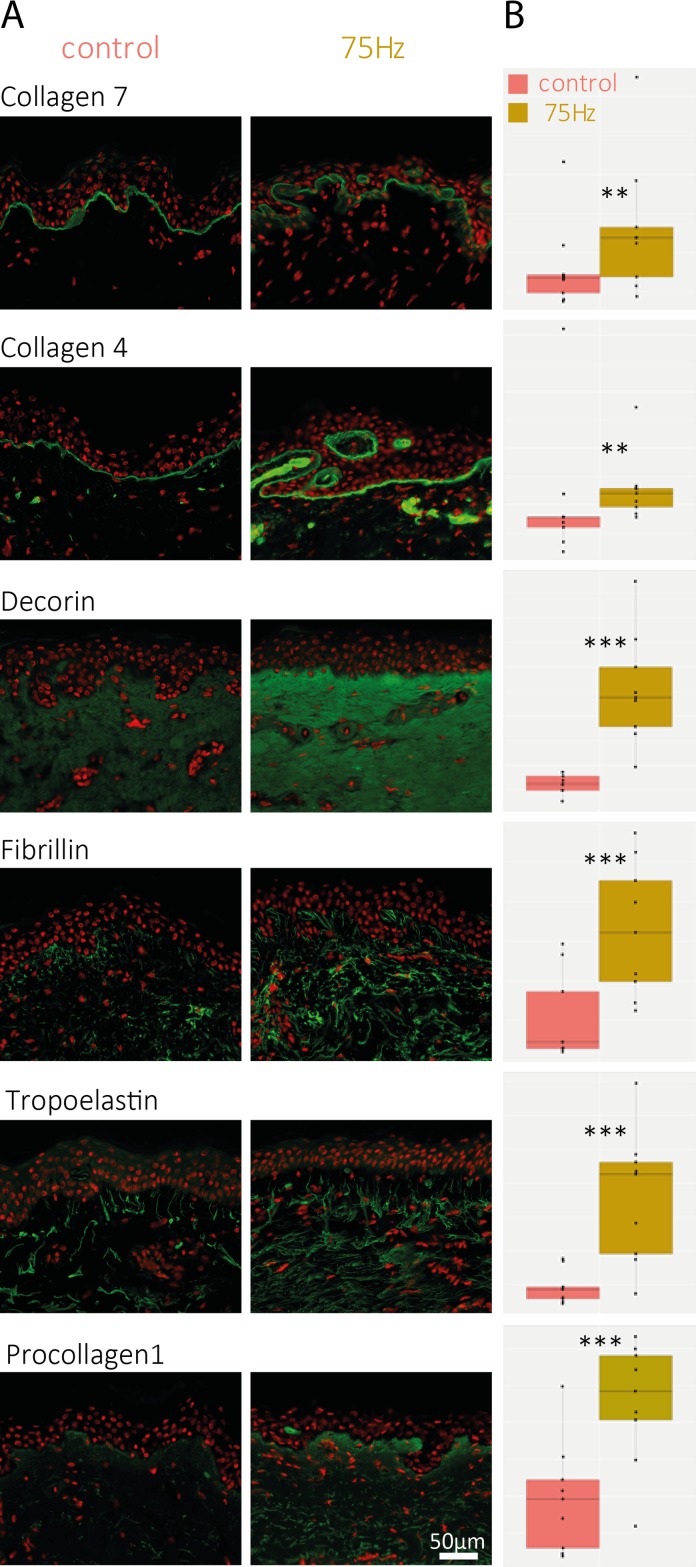
Immuno-labeling of DEJ and Dermal proteins for 75Hz treatment. (A) Immuno-labeling of some dermal markers (green labeling); (B)Box Plot representation of the fluorescence intensity (arbitrary scale) of the measured markers. Data shown are collected from one 68 year old donor, after 5 days of massaging for all markers, with the exception of type VII collagen and procollagen 1, which were sampled after 10 days of treatment from the same donor. A second 50 year old donor was analyzed with similar results (data not shown). The stars indicate the statistical significance of the labeling quantification for each condition compared to untreated/control skin (***: p<0.001, **: p<0.01 *: p<0.05).

Overall, explants massaged for ten days at 75Hz and 3° angular amplitude showed an increased expression of six proteins observed to varying extents at D5 and D10, as compared to their related controls (Fig 5). The increase of the collagen IV observed after treatment at 75Hz confirms the reinforcement of the DEJ observed in Fig 4 (with laminin 5 and perlecan markers). Moreover, in the dermis, and in particular in the papillary dermis, an important increase in decorin and procollagen 1 is observed, consistent with the fibronectin labeling shown before and with the structural changes observed in histological analysis (Fig 3). A strong increase of collagen VII, the anchoring fibril collagen, is observed in association with the increase of fibrillin and tropoelastin.

In brief, under our experimental conditions and at certain frequencies, massaging skin explants maintained in an acceptable survival biological state, clearly induces these tissues to respond to a rather soft mechanical impulse with an increased synthesis of certain skin structural proteins.

### In vivo

#### Effects of both regimens on clinical attributes

**Fig 6:**
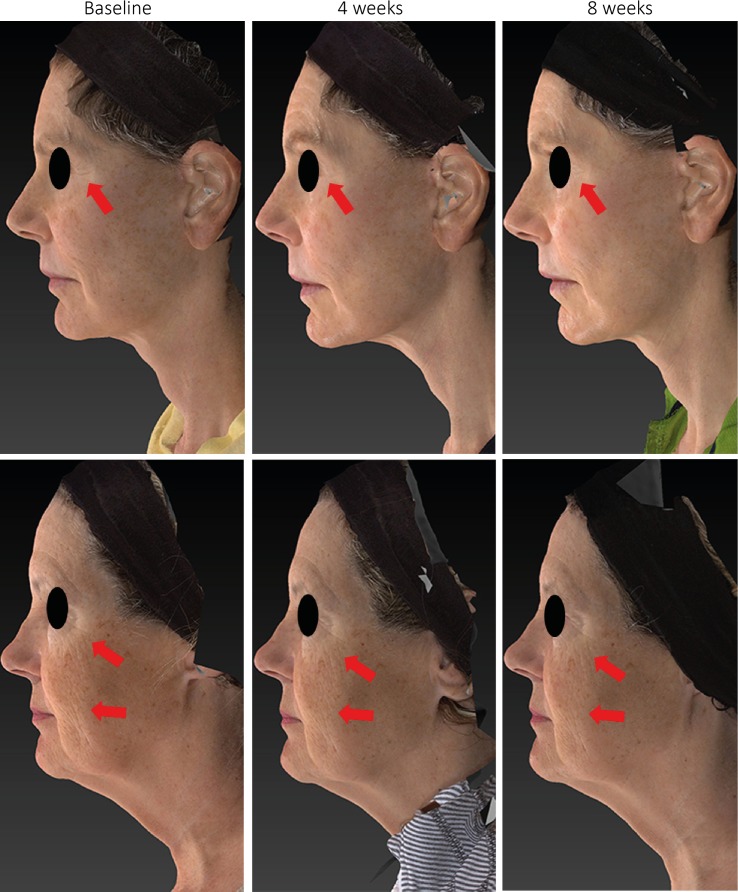
Global wrinkle changes during study. Example of two volunteers in Group 2 (cream plus sonic skin-massaging device) before (baseline), after 4 weeks of treatment and after 8 weeks of treatment. Standardized photographs illustrate an improvement of global facial wrinkles. Arrows point out specific areas of interest.

Both regimens (manual and device applications) significantly reduced the severity of several clinical attributes including global facial wrinkles, forehead wrinkles, skin texture, cheek wrinkles, and neck texture. The paired statistical comparisons between subgroups (Table 3) demonstrate a clear contribution from the massaging device in amplifying the effect of the cream at reducing the severity of global facial wrinkles skin texture, lip area wrinkles, cheek wrinkles, neck sagging, and neck texture. Fig 6 shows standardized photographs illustrating the changes to global wrinkles after 4 weeks and 8 weeks of treatment with the device compared with baseline.

**Table 3:** Paired comparisons between the two regimens. Paired comparisons between the cream + device and cream alone regimes expressed as differences in scores (scores at baseline minus scores at W4 and W8).

Attributes	Time point	Δ (Improvement from baseline) Device vs. Manual	Device vs. Manual
**Global facial wrinkles**	Week 4	0.31	p = 0.008
	Week 8	0.25	p = 0.049
**Texture**	Week 4	0.29	p = 0.008
	Week 8	0.23	p = 0.041
**Lip area wrinkles**	Week 4	0.23	p = 0.010
	Week 8	0.33	p = 0.155
**Cheek wrinkles**	Week 4	0.18	p = 0.155
	Week 8	0.31	p = 0.028
**Neck sagging**	Week 4	0.21	p = 0.013
	Week 8	0.28	p = 0.000
**Neck texture**	Week 4	0.26	p = 0.066
	Week 8	0.1	p = 0.099

Time of use (e.g. 4 or 8 weeks) apparently impacted the changes in scores of some features differently. Some of them appear more influenced by the duration (W4 to W8) of the use of the device (e.g. lip area or neck sagging) whereas others seem to plateau at W4.

## Discussion

The *ex vivo* study presented here demonstrates that an oscillatory mechanical stimulus exerted upon the skin surface can induce changes in the expression of some structural proteins, as previously shown in cellular models [1,8,40,41]. When the stimulation protocol is repeated twice per day, the effect is observed even when the stimulus is applied for short durations (1 min). Under these experimental conditions, some proteins appear to be expressed more than others; in particular the DEJ was reinforced (collagen IV, laminin V and perlecan) and the production of elastic fibers was increased (collagen VII, fibrillin, decorin, tropoelastin, fibronectin and procollagen 1). Massage treatment clearly induced some structural changes in the dermis, which appeared less degraded after 10 days of culture, compared to the control. Moreover, treatment at 75Hz induced some changes in the papillary dermis that could be related to the more intense immunolabeling signals observed in this area for fibronectin, decorin and procollagen 1. Although an additional study will be necessary to clarify their origin, the structural changes occurring in this area may be related to increased fibroblast activity provoked by the cyclic mechanical stimulation, which ultimately translates into increased ECM protein synthesis. Moreover, the increased expression of some proteins at the DEJ level, concomitant with the increased expression of elastin fibers, could support changes in the physical properties of the skin. A further study will be needed to investigate this possibility.

Protein expression appeared greater for cyclic mechanical stimuli around 75Hz. The mechanism underlying this remarkable result remains to be determined. Are some dermal proteins or proteoglycans more sensitive to frequency variance than others? Is an increased drainage of interstitial free water an important messenger of the mechanical stimulus for fibroblasts? The local mechanical stimulation seems to have a composite effect. On the one hand, local changes to the skin are observed in our *ex vivo* results, while on the other, more global effects are observed *in vivo* that could be related to the neuronal or endocrine system. In regenerative medicine, electrical stimuli are applied to a peripheral nerve to promote nerve regeneration [42]. The frequency of the electrical stimulation seems to be an important parameter. Hoffman demonstrated accelerated axon sprouting in partially denervated muscle in response to sinusoidal electrical stimulation between 50 and 100Hz [43]. The effects observed in our study occur in the same frequency range as that used by Hoffman. Is there a correlation between the frequency selectivity of the skin and the nervous system? The skin and the nervous system jointly exploit the immune system to provide additional signalling and regulatory input by using key stress mediators (such as corticotropin releasing hormone, ACTH, cortisol, nerve grow factor, etc)[23,24,44]. This pathway may explain why with only few seconds of local stimulation significant changes have been observed in the *in vivo* study presented here.

Despite these remaining questions, the *ex-vivo* procedure presented here provides a promising model for better understanding the biological effects of mechano-transduction at the tissue level.

*In vivo*, we observed that a commercial anti-wrinkle cream can lead to a significant decrease in the severity of some facial wrinkles and texture, as compared to baseline values (D0), in agreement with the claims made by the manufacturer, but had no effect on sagging. Importantly, using our massaging device in conjunction with the cream not only amplified the effects of the cream but also significantly reduced sagging (Table 3 and S1 Table). The questionnaires completed by the 20 subjects who used the massaging device indicated a total absence of side effects (itching, burning, edema, redness, etc.) and that the device was easy and comfortable to use. Most of the consumers using the massaging device perceived an improvement of fine lines, a smoother and healthier skin and a lifting effect after 4 weeks of treatment compared with subjects using the cream alone. In addition, there was also an improvement in wrinkle reduction and firmness of the skin after 8 weeks of treatment.

## Conclusion

The data presented here indicate that cyclic mechanical stimulation of *ex-vivo* human skin induces increased expression of certain DEJ and dermal proteins. Strikingly, protein expression was found to depend on the stimulus frequency and displayed a maximum around 75Hz. Such dynamic mechanical stimulation leads to amplification, *in vivo*, of the anti-wrinkle effect provided by the regular use of an anti-aging cosmetic regimen. Hence, with a device that delivers properly tuned stimuli, skin massaging provides an efficient anti-aging strategy. Future studies will help to determine the cellular and molecular mechanisms underlying our results.

## Supporting information

S1 Fig Positive and negative signal for decorin marker. Positive and negative signal for decorin marker for the control and the 75Hz treatment. Data shown are collected from one 68 year old donor, after 5 days of culture.
(TIF)

S1 Table Comparisons between the two regimens. Improvements, expressed as absolute differences (scores at baseline minus those at W4 and W8 for the two groups, cream + device and cream alone).
(DOCX)

S2 Table Study questionnaire immediate. Immediately after using the face cream and device test products, volunteers answered the following questions.
(DOCX)

S3 Table Study questionnaire immediate. Immediately after using the face cream test product, the volunteers answered the following questions.
(DOCX)

S4 Table Study questionnaire week 4 and 8. After using the face cream and device test products during 4 and 8 weeks, volunteers answered the following questions.
(DOCX)

S5 Table Study questionnaire week 4 and 8. After using the face cream test product during 4 and 8 weeks, volunteers answered the following questions.
(DOCX)

S1 Text Study protocol. The protocol of the clinical study is detailed here.
(DOCX)
