# Supplementary figures and images for: Effects of a skin-massaging device on the ex-vivo expression of human dermis proteins and in-vivo facial wrinkles
Source: PLoS One. 2017 Mar 1;12(3):e0172624. doi: 10.1371/journal.pone.0172624 (PMC5383004; doi:10.1371/journal.pone.0172624)

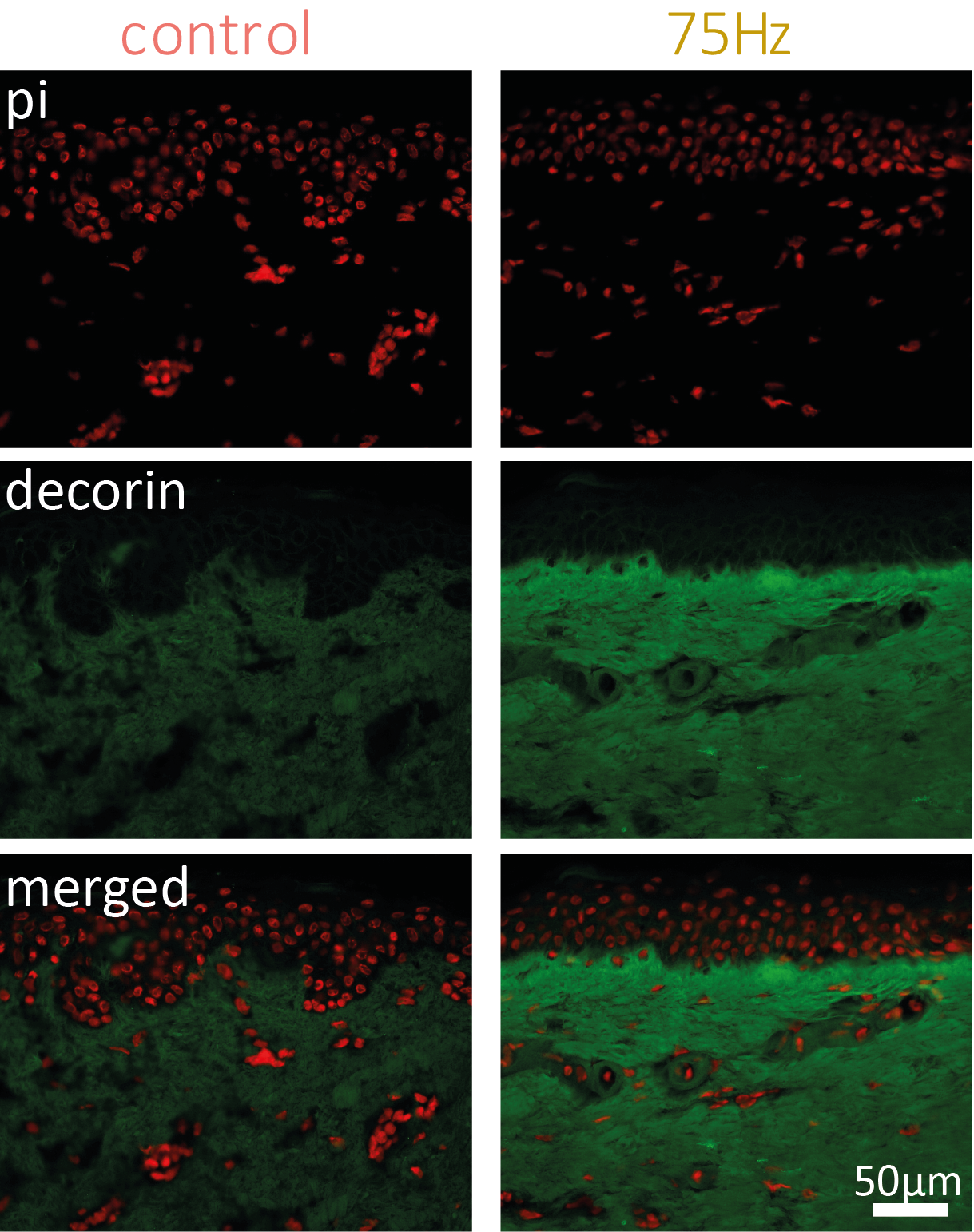

Supplement: S1 Fig — Positive and negative signal for decorin marker for the control and the 75Hz treatment. Data shown are collected from one 68 year old donor, after 5 days of culture. (TIF) [file pone.0172624.s001.tif]
