# Supplementary material for: Effects of a skin-massaging device on the ex-vivo expression of human dermis proteins and in-vivo facial wrinkles
Source: PLoS One. 2017 Mar 1;12(3):e0172624. doi: 10.1371/journal.pone.0172624 (PMC5383004; doi:10.1371/journal.pone.0172624)
Supplement: S1 Table — Improvements, expressed as absolute differences (scores at baseline minus those at W4 and W8 for the two groups, cream + device and cream alone). (DOCX) [file pone.0172624.s002.docx]

| **Attributes** | **Time point** | **Device Application** | | **Manual Application** | |
| --- | --- | --- | --- | --- | --- |
|  |  | **Δ (score difference with baseline) ±SD** |  | **Δ (score difference with baseline) ±SD** | **p-value vs. Baseline** |
| **Global facial wrinkles** | Week 4 | 0.63±0.36 | p<0.001 | 0.32±0.33 | p=0.026 |
|  | Week 8 | 0.73±0.38 | p<0.001 | 0.48±0.33 | p<0.001 |
| **Texture** | Week 4 | 0.65±0.29 | p<0.001 | 0.36±0.38 | p=0.022 |
|  | Week 8 | 0.73±0.34 | p<0.001 | 0.50±0.41 | p=0.000 |
| **Lip area wrinkles** | Week 4 | 0.50±0.40 | p=0.001 | 0.27±0.46 | p=0.194 |
|  | Week 8 | 0.60±0.42 | p<0.001 | 0.27±0.40 | p=0.152 |
| **Cheek wrinkles** | Week 4 | 0.50±0.40 | p=0.003 | 0.32±0.39 | p=0.036 |
|  | Week 8 | 0.65±0.46 | p<0.001 | 0.34±0.42 | p=0.026 |
| **Neck sagging** | Week 4 | 0.23±0.50 | p=0.256 | 0.02±0.11 | p=0.996 |
|  | Week 8 | 0.30±0.34 | p=0.068 | 0.02±0.11 | p=0.996 |
| **Neck texture** | Week 4 | 0.65±0.37 | p=0.000 | 0.39±0.49 | p=0.013 |
|  | Week 8 | 0.55±1.15 | p<0.001 | 0.45±0.53 | p=0.003 |
