# Supplementary material for: Effects of a skin-massaging device on the ex-vivo expression of human dermis proteins and in-vivo facial wrinkles
Source: PLoS One. 2017 Mar 1;12(3):e0172624. doi: 10.1371/journal.pone.0172624 (PMC5383004; doi:10.1371/journal.pone.0172624)
Supplement: S2 Table — Immediately after using the face cream and device test products, volunteers answered the following questions. (DOCX) [file pone.0172624.s003.docx]

| **Subject #**  **Initials:** | | | | | | | | |
| --- | --- | --- | --- | --- | --- | --- | --- | --- |
| **Please answer the following questions regarding the face cream that you used:** | | | | | | | | |
| **How much do you appreciate this face cream overall?** | Appreciate very much | Appreciate somewhat | | Neither appreciate nor do not appreciate | | Somewhat do not appreciate | | Do not appreciate at all |
| **What, if anything, did you LIKE about the application, feel of skin, and appearance of skin? Please be specific.** |  | | | | | | | |
| **What, if anything, did you DISLIKE about the application, feel of skin, and appearance of skin? Please be specific.** |  | | | | | | | |
| **How did the face cream apply on the skin on your face?** | Glided on skin very well | Glided on skin somewhat well | | Somewhat draggy on skin | | Very draggy on skin | |  |
| **Rate the speed of absorption of the face cream into the skin:** | Much too quickly | Somewhat too quickly | | Just right | | Somewhat too slowly | | Much too slowly |
| **Rate how moisturized/hydrated your skin feels with the face cream on:** | Very moisturized/ hydrated | Somewhat moisturized /hydrated | | Somewhat not moisturized/hydrated | | Not moisturized/ hydrated at all | |  |
| **Rate how comfortable your skin feels with the face cream on:** | Very comfortable | Somewhat comfortable | | Somewhat uncomfortable | | Very uncomfortable | |  |
| **If your skin felt uncomfortable, please explain why:** |  | | | | | | | |
| **Please answer the following questions regarding the applicator/device and face cream that you tested:** | | | | | | | | |
| **How much do you appreciate this applicator/device overall?** | Appreciate very much | Appreciate somewhat | | Neither appreciate nor do not appreciate | | Somewhat do not appreciate | | Do not appreciate at all |
| **What, if anything, did you LIKE about the applicator/device? Please be specific.** |  | | | | | | |  |
| **What, if anything, did you DISLIKE about the applicator/device? Please be specific.** |  | | | | | | |  |
| **Did the face cream/device irritate your skin at all?** | Yes | | | No | | | | |
| **If yes, please explain how your skin looked and/or felt irritated: (Be as specific as possible)** |  | | | | | | | |
| **Rate the improved appearance of fine lines after applying the face cream using the device:** | Very improved | | Somewhat improved | | Somewhat not improved | | | Not improved at all |
| **Rate the improved appearance of wrinkles after applying the face cream using the device:** | Very improved | | Somewhat improved | | Somewhat not improved | | | Not improved at all |
| **Rate the improved appearance of pores after applying the face cream using the device:** | Very improved | | Somewhat improved | | Somewhat not improved | | | Not improved at all |
| **Rate the improved appearance of skin firmness after applying the face cream using the device:** | Very improved | | Somewhat improved | | Somewhat not improved | | | Not improved at all |
| **Rate the improved appearance of saggy skin after applying the face cream using the device:** | Very improved | | Somewhat improved | | Somewhat not improved | | | Not improved at all |
| **Rate how youthful your skin appears after applying the face cream using the device:** | Very youthful | | Somewhat youthful | | Somewhat not youthful | | | Not youthful at all |
| **Rate how healthy your skin appears after applying the face cream using the device:** | Very healthy | | Somewhat healthy | | Somewhat not healthy | | | Not healthy at all |
| **Rate how smooth your skin appears after applying the face cream using the device** | Very smooth | | Somewhat smooth | | Somewhat not smooth | | | Not smooth at all |
| **Rate how radiant your skin appears after applying the face cream using the device:** | Very radiant | | Somewhat radiant | | Somewhat not radiant | | | Not radiant at all |
| **Rate how revitalized your skin appears after applying the face cream using the device** | Very revitalized | | Somewhat revitalized | | Somewhat not revitalized | | | Not revitalized at all |
| **Rate how much you agree with the following statement: The treatment gave skin a lifting effect** | Agree completely | | Agree somewhat | | Disagree somewhat | | | Disagree completely |
| **Please answer the following questions as they pertain to the applicator/device only:** | | | | | | | | |
| **Rate the feel of the applicator pad on your facial skin:** | Much too hard | | Somewhat too hard | | Just right | | Somewhat too soft | Much too soft |
| **Rate the size of the applicator pad:** | Much too large | | Somewhat too large | | Just right size | | Somewhat too small | Much too small |
| **Rate how well the applicator pad contours to your face:** | Very well | | Somewhat well | | Somewhat not well | | | Not well at all |
| **Rate the amount of vibration on your facial skin when using the applicator:** | Much too much | | Somewhat too much | | Just right amount | | Somewhat too little | Much too little |
| **Please answer the following questions as they pertain to your USUAL face cream usage:** | | | | | | | | |
| **Which method do you typically use to apply your face cream:** | Manually apply face cream | | | | Use a device to apply face cream | | | |
