# Supplementary material for: Effects of a skin-massaging device on the ex-vivo expression of human dermis proteins and in-vivo facial wrinkles
Source: PLoS One. 2017 Mar 1;12(3):e0172624. doi: 10.1371/journal.pone.0172624 (PMC5383004; doi:10.1371/journal.pone.0172624)
Supplement: S5 Table — After using the face cream test product during 4 and 8 weeks, volunteers answered the following questions. (DOCX) [file pone.0172624.s006.docx]

| **Subject #**  **Initials:** | | | | | | | | | | | | |
| --- | --- | --- | --- | --- | --- | --- | --- | --- | --- | --- | --- | --- |
| **Please answer the following questions regarding the face cream that you used:** | | | | | | | | | | | | |
| **How much do you appreciate this face cream overall?** | Appreciate very much | Appreciate somewhat | | | Neither appreciate nor do not appreciate | | | | | Somewhat do not appreciate | | Do not appreciate at all |
| **What, if anything, did you LIKE about the application, feel of skin, and appearance of skin? Please be specific.** |  | | | | | | | | | | | |
| **What, if anything, did you DISLIKE about the application, feel of skin, and appearance of skin? Please be specific.** |  | | | | | | | | | | | |
| **How did the face cream apply on the skin on your face?** | Glided on skin very well | Glided on skin somewhat well | | | Somewhat draggy on skin | | | | | Very draggy on skin | |  |
| **Rate the speed of absorption of the face cream into the skin:** | Much too quickly | Somewhat too quickly | | | Just right | | | | | Somewhat too slowly | | Much too slowly |
| **Rate how moisturized/hydrated your skin feels with the face cream on:** | Very moisturized/ hydrated | Somewhat moisturized /hydrated | | | Somewhat not moisturized/hydrated | | | | | Not moisturized/ hydrated at all | |  |
| **Rate how comfortable your skin feels with the face cream on:** | Very comfortable | Somewhat comfortable | | | Somewhat uncomfortable | | | | | Very uncomfortable | |  |
| **If your skin felt uncomfortable, please explain why:** |  | | | | | | | | | | | |
| **Did the face cream irritate your skin at all?** | Yes | | | | | | No | | | | | |
| **If yes, please explain how your skin looked and/or felt irritated: (Be as specific as possible)** |  | | | | | | | | | | | |
| **Please rate how much you agree or disagree with the following statements:** | | | | | | | | | | | | |
| **The product texture is…** | Completely agree | | | Somewhat agree | | | | Somewhat disagree | | | | Completely disagree |
| Pleasant |  | | |  | | | |  | | | |  |
| Smooth |  | | |  | | | |  | | | |  |
| Creamy |  | | |  | | | |  | | | |  |
| **Please rate the consistency of the test product. Did you find it to be…** | Much too thick | | Somewhat too thick | | | Just about right consistency | | | Somewhat too thin | | Much too thin | |
| **Did you notice if the test product had a scent?** | yes | | | | | | | no | | | | |
| **Please rate how much you agree or disagree with the following statements:** | | | | | | | | | | | | |
| **The scent of the product is…** | Completely agree | | | Somewhat agree | | | | Somewhat disagree | | | | Completely disagree |
| Pleasant |  | | |  | | | |  | | | |  |
| Appropriate for face cream |  | | |  | | | |  | | | |  |
| **Please rate the strength/intensity of the scent. Did you find it to be:** | Much too strong | | Somewhat too strong | | | Just about right | | | Somewhat too weak | | Much too weak | |
| **Please rate how much you agree or disagree with the following statements:** | | | | | | | | | | | | |
| **The face cream feels ________ on my skin…** | Completely agree | | | Somewhat agree | | | | Somewhat disagree | | | | Completely disagree |
| Light/not heavy |  | | |  | | | |  | | | |  |
| Pleasant |  | | |  | | | |  | | | |  |
| Smooth |  | | |  | | | |  | | | |  |
| Soft |  | | |  | | | |  | | | |  |
| Fresh/refreshing |  | | |  | | | |  | | | |  |
| **Rate how greasy/not greasy your skin feels with the facial moisturizer on. Does it feel….** | Not greasy at all | | | Slightly greasy | | | | Moderately greasy | | | | Very greasy |
| **Is the level of greasiness…** | Acceptable | | | | | | | Unacceptable | | | | |
| **Please rate how much you agree or disagree with the following statements:** | | | | | | | | | | | | |
| **My skin appears __________ with the face cream on…** | Completely agree | | | Somewhat agree | | | | Somewhat disagree | | | | Completely disagree |
| Even-toned |  | | |  | | | |  | | | |  |
| Radiant |  | | |  | | | |  | | | |  |
| Healthy |  | | |  | | | |  | | | |  |
| Smooth |  | | |  | | | |  | | | |  |
| Fresh/refreshed |  | | |  | | | |  | | | |  |
| Non-greasy |  | | |  | | | |  | | | |  |
| **Rate the improved appearance of fine lines after applying the face cream** | Very improved | | | Somewhat improved | | | | Somewhat not improved | | | | Not improved at all |
| **Rate the improved appearance of wrinkles after applying the face cream** | Very improved | | | Somewhat improved | | | | Somewhat not improved | | | | Not improved at all |
| **Rate the improved appearance of pores after applying the face cream using the device:** | Very improved | | | Somewhat improved | | | | Somewhat not improved | | | | Not improved at all |
| **Rate the improved appearance of skin firmness after applying the face cream** | Very improved | | | Somewhat improved | | | | Somewhat not improved | | | | Not improved at all |
| **Rate the improved appearance of saggy skin after applying the face cream** | Very improved | | | Somewhat improved | | | | Somewhat not improved | | | | Not improved at all |
| **Rate how youthful your skin appears after applying the face cream** | Very youthful | | | Somewhat youthful | | | | Somewhat not youthful | | | | Not youthful at all |
| **Rate how healthy your skin appears after using the face cream** | Very healthy | | | Somewhat healthy | | | | Somewhat not healthy | | | | Not healthy at all |
| **Rate how smooth your skin appears after using the face cream** | Very smooth | | | Somewhat smooth | | | | Somewhat not smooth | | | | Not smooth at all |
| **Rate how radiant your skin appears after using the face cream** | Very radiant | | | Somewhat radiant | | | | Somewhat not radiant | | | | Not radiant at all |
| **Rate how revitalized your skin appears after using the face cream** | Very revitalized | | | Somewhat revitalized | | | | Somewhat not revitalized | | | | Not revitalized at all |
| **Rate how much you agree with the following statement: Face cream gave skin a lifting effect** | Agree completely | | | Agree somewhat | | | | Disagree somewhat | | | | Disagree completely |
