# Supplementary material for: Effects of a skin-massaging device on the ex-vivo expression of human dermis proteins and in-vivo facial wrinkles
Source: PLoS One. 2017 Mar 1;12(3):e0172624. doi: 10.1371/journal.pone.0172624 (PMC5383004; doi:10.1371/journal.pone.0172624)
Supplement: S1 Text — The protocol of the clinical study is detailed here. (DOCX) [file pone.0172624.s007.docx]

**Study Title:** Evaluating the efficacy of a prototype massage applicator using a prototype cream and manual application of a prototype cream on a population with signs associated with aging skin

**Compliance Statement**

The protocol was approved by the Western Institutional Review Board (WIRB: Olympia, WA USA) following FDA and ICH guidelines. Written and informed consent was obtained from all subjects prior to enrollment.

**Study Endpoints**

*Efficacy Endpoints*

- Clinical grading at baseline, week 4, and week 8:
  - Face: Forehead wrinkles, cheek wrinkles, lip area wrinkles, global facial wrinkles, facial sagging, glabella lines, texture
  - Neck: Overall appearance, sagging, neck folds, texture
- Digital imaging procedures: Vectra 3D Imaging System, Canfield
- Subject completed self-assessment questionnaires (week 4 and week 8).

*Safety/Tolerability Endpoints*

- Clinical grading of objective irritation parameters and subject reporting of subjective assessment parameters at baseline, week 4, and week 8.
- Monitoring of adverse events throughout the course of the study.

Overview

| **Study Title** | Evaluating the efficacy of a prototype massage applicator using a prototype cream and manual application of a prototype cream on a population with signs associated with aging skin |
| --- | --- |
| **Sample Size** | 45 enrolled to complete 42 |
| **Study Design** | - 2 arms:   - tagged prototype massage applicator using a sonic handle and prototype cream   - Manual application of prototype cream - Subjects randomized to a treatment application method (prototype massage applicator + prototype cream, manual application of prototype cream) - Home use: two times per day; 7 days per week   - Prototype massage applicator using prototype cream for 2 minutes and 15 seconds (30 seconds forehead, 1 minute mouth and cheek area, and 45seconds jawline and neck)   - Manual application of facial cream (using their normal technique for applying facial creams) - Duration: 8 weeks - Study visits: Baseline, 4 and 8 weeks - Clinical assessments: Baseline, 4 and 8 weeks - Self-assessment questionnaires: 4 and 8 weeks   A sunscreen (SPF 30) will be given to subjects at the baseline visit for daily use (mornings only) and to be reapplied during the day as needed for the duration of the study |
| **Number of Arms** | 2 arms:   - Prototype massage applicator using a sonic handle and prototype cream - Manual application of prototype cream |
| **Test Sites** | - Face - Neck |
| **Key entrance criteria** | - Available for 3 study visits - Between the ages of 65-75 years - Have not participated in a study evaluating Anti-aging applicators/devices - Fitzpatrick skin types I, II, III - Non-smokers - Fine lines/wrinkles: global facial wrinkles ( ≥ 4 on a 10 pt. scale) - Sagging facial skin ( ≥4 on a 10 pt. scale) |
| **Parameters** | - Photographs at baseline, 4, & 8 weeks:   - Vectra 3D Imaging System - Expert/Esthetician clinical grading (face/neck):   - Face: Forehead wrinkles, cheek wrinkles, lip area wrinkles, global facial wrinkles, facial sagging, glabella lines, texture   - Neck: Overall appearance, sagging, neck folds, texture - Tolerance (0 – 3 point assessment scale):   - Objective tolerance: erythema, edema, dryness and peeling   - Subjective tolerance: burning, stinging, itching and tingling - Self-assessment questionnaires (4 & 8 week) |
| **Number of Visits/Study Duration** | - 4 visits/8 weeks |
| **Supplies needed** | - 23: handle - 23: Anti-aging massage applicator (RFID tagged) - 115: 2 oz jars Prototype cream   - 3 jars per subject for the device arm of the study   - 2 jars per subject for the manual arm of the study - 100: plastic spatulas - 45: SPF 30 sunscreen (4.2 oz tubes) - Cameras: Vectra - 45: Travel bags |
| **Visit Summary** | *Baseline (Visit 1):*   - 15 minute acclimation period - Study & photograph consent forms signed - Medical History reviewed - Assign study number to the subject - The examiner blinded to the application method will conduct the following:   - Tolerance (0 – 3 point scale):     - Objective tolerance: erythema, edema, dryness and peeling     - Subjective tolerance: burning, stinging, itching and tingling   - Clinical grading assessments on the face and neck (0-9 point scale):     - Face:       - forehead wrinkles       - cheek wrinkles       - lip area wrinkles       - global facial wrinkles       - facial sagging       - glabella lines       - texture     - Neck:       - overall appearance       - sagging       - neck folds       - texture - Photos prior to product use: Vectra - Study personnel not blinded to the application method will randomize the subject to a treatment group - Study personnel not blinded to the application method will train the subject on product use:   - Have subjects examine their face and neck in a mirror prior to product use with study personnel pointing out areas to follow for the duration of the study; fine lines and wrinkles, sagging, texture, neck folds, glabella lines, overall appearance, etc.   - For subjects randomized to the *manual application groups*:     - No instructions on how to or how long or how much facial cream to apply the face and neck will be given. Subjects will be instructed to apply the study cream using the amount of cream and technique of application they normally use at home when applying a facial cream     - Subject applies the study cream to the face and neck using the amount of cream and technique they normally use at home to apply facial creams     - Subjects will be instructed to apply the study cream twice per day (morning and bedtime)   - For subjects randomized to the prototype device group:     - Subjects will be trained on how to use prototype device     - The study personnel will explain and demonstrated to the subjects how much cream to use with the anti-aging applicator and how to apply the cream to the treatment zones (refer to Volunteer Instructions)     - Subjects applies the prototype study cream to each treatment zone (forehead, cheeks/mouth and neck/jawline) for a total of 2 minutes and 15 seconds using the prototype device /prototype cream for the following times per treatment zone:       - Zone 1: forehead - 30 seconds       - Zone 2: cheeks and mouth area - 1 minute       - Zone 3: neck and jawline – 45 seconds     - Discontinue all current serum, moisturizer, and/or any other skincare products     - Subjects will be instructed to use the study products twice per day (morning and bedtime) - Remind subjects to bring all study products to next study visit - Instruct subjects to discontinue using their current serum, moisturizer, and/or any other skin care products and replace with study products - A sunscreen SPF 30 will be given to the subjects with instructions to apply the sunscreen every morning and to reapply throughout the day as needed   *Visit 2 & 3 [Visit 2[4 weeks (28 days ±2 days) post baseline], Visit 3 [8 weeks (56 days ±2 days) post baseline]*   - 15 minute acclimation period - Review medical history and discuss product use - Document adverse events and protocol deviation as indicated - Confirm subject has applied the study products per application method at least 2 hours prior to the study visit. - Confirm subject has no makeup, skin care products (eye products/eye makeup permitted) including sunscreen on the face and neck the morning of the study visit (besides study cream) - The examiner blinded to the application method will conduct the following:   - Tolerance (0 – 3 point scale):     - Objective tolerance: erythema, edema, dryness and peeling     - Subjective tolerance: burning, stinging, itching and tingling   - Clinical grading assessments on the face and neck (0-9 point scale):     - Face:       - forehead wrinkles       - cheek wrinkles       - lip area wrinkles       - overall facial wrinkles       - facial sagging       - glabella lines       - texture     - Neck:       - overall appearance       - sagging       - neck folds       - texture - Photos prior to product use: Vectra - Subject completes a self-assessment questionnaire - Additional product dispensed if needed (Visit 2 only)   Study products collected and subject compensated for participation (Visit 3 only) |
|  |  |
|  |  |

# **Volunteer Selection**

A. Population:

45 volunteers will be enrolled in this 8 week study to complete 42.

B. Enrollment Criteria:

*Inclusion criteria:*

Volunteers meeting the following criteria will be enrolled:

- Between the ages of 65-75 years;
- Available for 3 study visits;
- Have not participated in a study evaluating anti-aging applicators/devices;
- Fitzpatrick skin types I, II, III, IV
- Fine lines/wrinkles: global facial wrinkles ( ≥ 4 on a 10 pt. scale);
- Sagging facial skin ( ≥ 4 on a 10 pt. scale);
- Individuals who are non-smokers;
- Individuals that are willing to provide written informed consent and are able to read, speak, write, and understand English;
- Individuals willing not to wear makeup or use skin care products other than the study products the day of the study visits;
- Individuals willing to discontinue using current skincare products and only use study products for the study duration
- Individuals willing to refrain from sun tanning / sun bathing for the study duration.

*Exclusion criteria:*

Volunteers will be excluded from the study if they meet any of the criteria listed below:

- Present or disclose any medically diagnosed chronic skin problems on their face (e.g., psoriasis, eczema, seborrheic dermatitis, or chronic cystic acne, etc.)
- Diagnosed with known allergies to facial skin care products
- Have undergone dermatological skin rejuvenation procedures such as light-therapies (IPL, lasers, radiofrequency and other lights treatments ) or such as collagen or other facial tissue augmentation, fillers, or retail microdermabrasion on the face within the last 1 year preceding the baseline visit and intended to receive them during the study period
- Have undergone Botox treatment on the face within the last 6 months preceding the baseline visit and intend to receive them during the study period
- Have a history of immunosuppression/immune deficiency disorders (including HIV infection or AIDS) or currently using immunosuppressive medication (e.g., azathioprine, belimumab, cyclophosphamide, Enbrel, Imuran, Humira, mycohenolate mofetil, methotrexate, prednisone, Remicade, Stelara)
- Have uncontrolled disease such as diabetes, hypertension, hyperthyroidism, or hypothyroidism. Individuals having multiple health conditions may be excluded from participation even if the conditions are controlled by diet, medication, etc.
- Have planned surgeries or invasive procedures during the course of the study.
- Have hypersensitive skin to chemical agents.
- Excessively tanned face before the baseline visit and who intend to excessively be exposed to sun or artificial U. V. during the study period
- Have hypersensitive skin to chemical agents
- Have sunburned skin
- Are an employee of Clarisonic

**Volunteer Instructions**

Subjects will be instructed not to wear any facial makeup or apply any skin care products or sunscreen on the face and neck on the day of the baseline, 4 and 8 week study visits. At the day of the 4 and 8 week visits will also be instructed to apply the study facial cream at least 2 hours prior to the study visits. They will also be instructed to discontinue using all of their current skin care products such as serums and moisturizers except the study product for the duration of the study. The study anti-aging cream (applied manually using their normal technique or using the prototype massage applicator) will be used at home twice per day for the 8 week duration of the study.

**Procedures**

1. Evaluation Procedures:

*Recruiting:*

- Potential volunteers will be contacted via a recruiting database
- Interested volunteers meeting the online screening criteria will be scheduled for a baseline visit.

*Pre-Study Instructions:*

The day of the baseline visit subjects will be instructed not to use apply any makeup, sunscreen, or moisturizer on their face and neck for at least 2 hours prior to the study visit.

B. Outline of Procedures:

| **Procedures** |  |  |  |
| --- | --- | --- | --- |
|  | Baseline | 4 Week | 8 Week |
| No facial skincare products including makeup, moisturizer, and sunscreen on face and neck at least 2 hours before the study visits | X |  |  |
| Informed consent, medical history, entrance criteria | X |  |  |
| 15 minute acclimation period | X | X | X |
| No skincare products (makeup, sunscreen) besides the study cream prior to the study visits (eye makeup permitted) |  | X | X |
| Confirm subject has used the study products at least 2 hours prior to the study visit (no sunscreen) |  | X | X |
| Esthetician Tolerance Assessment  Erythema Dryness  Peeling Edema | X | X | X |
| Subject Tolerance Assessment  Stinging Itching  Tingling Burning | X | X | X |
| Esthetician Efficacy Assessment  Face (forehead, cheek, lips, global face, sagging. Glabella lines, texture)  Neck (overall appearance, sagging, neckfolds, texture) | X | X | X |
| Photography  -Vectra | X | X | X |
| Volunteer Instruction distribution | X |  |  |
| Product Distribution/Return***** | D | D** | R |
| Subject randomized to treatment & trained | X |  |  |
| Subject Questionnaire |  | X | X |
| Subject compensation |  |  | X |

***D=dispense; R= Return**

**** only if additional product is needed**

C. Assessments:

*Clinical grading assessments:*

Clinical grading of efficacy parameters will be performed at baseline, 4 & 8 weeks. Subjects will be provided with a black headband to keep hair away from the face and instructed on proper headband placement. Full face and neck efficacy parameters will be assessed on each subject using the modified Griffiths’ 10-point scale recording to the following numerical definitions (half-point scores may be used as necessary to more accurately describe the skin condition):

0 = none (best possible condition)

1 to 3 = mild

4 to 6 = moderate

7 to 9 = severe (worst possible condition)

The following parameters to assess the **face** will be evaluated using the indicated scale anchors:

| Parameter | Location | 0 = | 9 = |
| --- | --- | --- | --- |
| Facial sagging (global) | Overall | None | Severe sagging |
| Texture | Overall | Even | Severely rough and uneven skin |
| Fine lines/wrinkles  Glabella lines | Forehead, cheek, lip, global face  Forehead | None    None | Numerous, deep wrinkles  Numerous, deep lines |

The following parameters to assess the **neck** will be evaluated using the indicated scale anchors:

| Parameter | Location | 0 = | 9 = |
| --- | --- | --- | --- |
| Sagging (global) | Overall | None | Severe sagging |
| Neck folds  Texture  Overall appearance | Overall  Overall  Overall | None    Even  Very healthy | Numerous, deep folds  Severely rough and uneven skin  Very unhealthy |

*Tolerance assessments:*

Tolerability evaluations will be performed at baseline, weeks 4 and 8. Local cutaneous tolerability will be evaluated by assessing the overall signs and symptoms of erythema, edema, dryness, and peeling and by the subject reporting the overall degree of burning, stinging, itching, and tingling.

For overall assessments of subjective irritation, subjects will report the degree of any parameters that they typically experience when using a product similar to the test material(s) at the baseline visit. At post-baseline time points, subjects will report the degree of any of these symptoms they have experienced since the previous time point.

The following static (without reference to any prior visits) scales and definitions will be used for overall tolerability evaluations (with half-point scores used as necessary to better describe the clinical condition):

Objective Irritation Parameters:

| Erythema | |
| --- | --- |
| 0 = None | No erythema of the treatment area |
| 1 = Mild | Slight, but definite redness of the treatment area |
| 2 = Moderate | Definite redness of the treatment area |
| 3 = Severe | Marked redness of the treatment area |

| Edema | |
| --- | --- |
| 0 = None | No edema/swelling of the treatment area |
| 1 = Mild | Slight, but definite edema of the treatment area |
| 2 = Moderate | Definite edema of the treatment area |
| 3 = Severe | Marked edema of the treatment area |

| Dryness | |
| --- | --- |
| 0 = None | No dryness of the treatment area |
| 1 = Mild | Slight, but definite dryness of the treatment area |
| 2 = Moderate | Definite dryness of the treatment area |
| 3 = Severe | Marked dryness of the treatment area |

| Peeling | |
| --- | --- |
| 0 = None | No peeling of the treatment area |
| 1 = Mild | Slight, but definite peeling of the treatment area |
| 2 = Moderate | Definite peeling of the treatment area |
| 3 = Severe | Marked peeling of the treatment area |

*Subjective Irritation Parameters*:

| Burning | |
| --- | --- |
| 0 = None | No burning of the treatment area |
| 1 = Mild | Slight burning sensation of the treatment area; not really bothersome |
| 2 = Moderate | Definite warm, burning of the treatment area that is somewhat bothersome |
| 3 = Severe | Hot burning sensation of the treatment area that causes definite discomfort and may interrupt daily activities and/or sleep |

| Stinging | |
| --- | --- |
| 0 = None | No stinging of the treatment area |
| 1 = Mild | Slight stinging sensation of the treatment area; not really bothersome |
| 2 = Moderate | Definite stinging of the treatment area that is somewhat bothersome |
| 3 = Severe | Marked stinging sensation of the treatment area that causes definite discomfort and may interrupt daily activities and/or sleep |

| Itching | |
| --- | --- |
| 0 = None | No itching of the treatment area |
| 1 = Mild | Slight itching sensation of the treatment area; not really bothersome |
| 2 = Moderate | Definite itching of the treatment area that is somewhat bothersome |
| 3 = Severe | Marked itching sensation of the treatment area that causes definite discomfort and may interrupt daily activities and/or sleep |

| Tingling | |
| --- | --- |
| 0 = None | No skin tingling sensation of the treatment area |
| 1 = Mild | Slight, but definite tingling sensation of the treatment area |
| 2 = Moderate | Definite tingling sensation of the treatment area that is somewhat bothersome |
| 3 = Severe | Marked tingling sensation of the treatment area that causes definite discomfort |

1. Imaging Procedures

Prior to photography procedures, clinic personnel will ensure that subjects have a clean face with no makeup as described in the study procedures. Subjects will remove any jewelry from the area(s) to be photographed and acclimatize for at least 15 minutes to ambient conditions within the clinic before any photographs are taken. Subjects will be provided with a black headband to keep hair away from the face and instructed on proper headband placement. Subjects will be provided with a black matte cloth that will be draped over the subjects’ clothing.

Images will be taken with the Vectra camera to capture volumetric images. Every day prior to taking photographs the camera will be calibrated. All photos will be taken with one frontal view at baseline, week 4, and week 8. Positioning of the subject’s posture is crucial to ensure proper volumetric measurements. The subject must sit in an erect, upright posture with a relaxed facial expression emphasizing that the teeth slightly touch with a relaxed jaw (no clenching). Alignment of the facial position at varying time points is also crucial to quantify volumetric data.

1. Self-Assessment Questionnaires:

Subjects will complete a self-assessment questionnaire at baseline, week 4 and week 8. Questionnaires will be provided by the Consumer Evaluation group.

1. Study Procedures:
   The study researchers will conduct the following procedures:

*The following sequence of procedures will be conducted:*

| **Prescreening:** | | |
| --- | --- | --- |
| *Sequence:* | *Procedure* | *Conducted by:* |
| 1 | Potential volunteers will be contacted via a recruiting database | Study Coordinator |
| 2 | Interested volunteers meeting the criteria via online qualification questionnaire will be scheduled for study visits | Study Coordinator /volunteer |
| **Baseline visit:** | | |
| *Sequence:* | *Procedure:* | *Conducted by:* |
|  | WIRB approved Study Consent and Photograph Release forms signed and the subject acclimatizes for 15 minutes | Subject |
|  | Medical history reviewed and updated, Inclusion/exclusion criteria checklist reviewed and verified, and subject number assigned | Blinded Study Esthetician |
|  | Subject completes online baseline questionnaire | Blinded Study Esthetician |
|  | Confirm the subject has no makeup, skin care products including moisturizer and sunscreen on the face and neck (eye makeup/eye products permitted) at least 2 hours prior to the study visit. | Blinded Study Esthetician |
|  | The following tolerance & clinical grading assessments are conducted:   - Objective/Subjective tolerance assessments - Clinical Grading:   - *Face:*     - Forehead- fine lines/wrinkles     - Cheeks- fine lines/wrinkles     - Lips – fine lines/wrinkles     - Global - fine lines/wrinkles     - Facial sagging (global)     - Texture     - Glabella lines   - *Neck:*     - Overall appearance     - Sagging     - Neck folds     - Texture | Blinded Study Esthetician |
|  | Photos: Vectra | Photographer |
|  | Identify areas that the subject will follow for changes in their skin via the aid of a mirror | Unblinded Study Personnel |
|  | The subject will be given the following:   - Study instructions - Randomized to a treatment group - Dispense Volunteer Instructions and study product | Unblinded Study Personnel |
|  | The subject will trained on how to use the study products (only device group) | Unblinded Study Personnel |
|  | The subject is instructed to use the study products according the Volunteer Instructions sheet. The instructions will be reviewed step by step with each subject | Unblinded Study Personnel |
|  | The subject is reminded to bring all study products to next study visit | Coordinator |
| ***Visits 2 & 3 (4 & 8 week ±2 days study visits):*** | | |
|  | The subject acclimates for 15 minutes | Blinded Study Esthetician |
|  | Review medical history and document adverse events and protocol deviation as indicated. Confirm the subject has no makeup or sunscreen on face and has used study products at least 2 hours before appointment | Blinded Study Esthetician |
|  | Confirm the subject has used the study products on the face and neck twice per day (morning & bedtime). | Blinded Study Esthetician |
|  | The following clinical grading & tolerance assessments are conducted:   - Objective/Subjective tolerance assessments - Clinical grading assessments:   - *Face:*     - Forehead- fine lines/wrinkles     - Cheeks- fine lines/wrinkles     - Lips – fine lines/wrinkles     - Global - fine lines/wrinkles     - Facial sagging (global)     - Texture     - Glabella lines   - *Neck:*     - Overall appearance     - Sagging     - Neck folds     - Texture | Blinded Study Esthetician |
|  | Photos: Vectra | Photographer |
|  | Questionnaire completed | Subject |
|  | Additional study anti-aging cream dispense, if needed (Visit 2 only) | Blinded Study Esthetician |
|  | The subject is reminded to bring all study products to next study visit. The date of the next study visit verified and confirmed (Visit 2 only) | Study Coordinator |
|  | Study product collected (Visit 3 only)  The subject is compensated for participation (Visit 3 only) | Blinded Study Esthetician |

# **Study endpoint**

# There is a statistically/clinically significant reduction in the study parameters between the subjects randomized to the Anti-aging applicator with prototype cream and the subjects randomized to manual application of the prototype cream.

# **Adverse Events**

Subjects will be examined at each study visit to determine if they experienced any adverse events. Any adverse events will be reported and an assessment will be made of the relationship of the reaction to the study product by the Principal Investigator and recorded as “probably related”, “possibly related”, “unrelated”, or “unknown”.

Adverse events related to the study will be documented at each study visit and will be recorded and handled according to the regulations.

**Study records**

Study records including study related documents pertaining to the conduct of the study will be kept in a secure area. Patient information will remain confidential.

**Safety**

All products used in this study will be reviewed for safety and biocompatibility as per standard operation procedures.

**Statistical Analysis**

The clinical grading scores and tolerance scores (subjective and objective) will be compared for both treatments and with their respective baselines: Anti-aging applicator + prototype cream and manual application of prototype cream at all the time points (week 4 and week 8).

For comparison with baseline of each treatment, the normality of the difference (from baseline) will be tested with Shapiro-Wilk test. The following statistical analyses will be performed for comparisons:

- Parametric Paired t-test in case of normality of differences
- Non-parametric Wilcoxon Signed Ranks test in the inverse case

For comparing both the treatments with each other, the normality of the difference (between treatments) will be tested with Shapiro-Wilk test. The following statistical analyses will be performed for comparisons:

- Parametric t-test in case of normality of differences
- Non-parametric Mann-Whitney test in the inverse case

For normality tests, the statistical significance level is set at p≤0.01. For all other statistical tests, the statistical significance level is set at p≤0.05.

Clinical significance is equal to or greater than a half point difference in clinical grading scores. Clinical significance will be addressed only when the data is statistically significant.

The self-assessment data will be analyzed by a consumer perception team.

Final Report

Following the completion of the study, a final report will be prepared. The report will detail the number of subjects who completed the study, any protocol deviations, adverse events, modifications, and a summary of the data.

**Volunteer Instructions**

**VOLUNTEER INSTRUCTIONS**

**Manual Group**

Pacific Bioscience Labs, Inc. would like to thank you for participating in this randomized, parallel arm home use test to evaluate signs associated with aging. You have been randomized to the manual application of the study facial cream group. The purpose of this evaluation is to assess the effectiveness in treating signs associated with aging of a prototype massage applicator in applying a facial cream compared to manual application of a facial cream over an 8 week period.

You will be given a study facial cream to use at home twice per day (morning and bedtime) for the next 8 weeks.

After using the facial cream for the first time and at each study visit you will complete an online questionnaire. In order to adequately complete the study questionnaires, please pay close attention to the following:

- Appearance of overall facial fine lines/wrinkles as well as the fine lines and wrinkles on the forehead, cheek, and upper lip/mouth
- Appearance of skin sagging on the face and neck areas
- Appearance of the lines between your eyes/eyebrows
- Overall appearance of the skin on your neck and neck folds
- Overall texture of the skin on your face and neck

For the next 8 weeks you will be asked to cleanse your face twice per day (approximately the same time each morning and evening prior to bedtime) using the cleanser and cleansing method you normally use. After cleansing, rinse your face with warm water, pat dry with a towel, and apply the study cream using the amount and technique you normally use when applying a facial cream. Please apply the study facial cream to your face and neck. Please do not change any other skin care routines except adding the study cream to your routine.

**Remember: You are asked to discontinue using your current facial moisturizer, serum and/or any other facial product, but please do not change cleaning method, skin care cleansing products for the study duration.**

You will be instructed to:

- Discontinue using your current facial moisturizer, serum, and/or any other facial skin care products
- Use the study facial cream twice per day (morning and bedtime) on your face and neck area
- Apply the study sunscreen to the face and neck (morning only). Please avoid contact of the sunscreen with the eyes. In the event the sunscreen comes in contact with the eye, rinse the eye with water to remove.
- Closely examine your face and neck prior to and after using study facial cream

**Protocol for the day of each study visit:**

- Morning of visits: Cleanse your face using your normal cleanser and cleansing method (approximately same time each morning)
- Apply the study facial cream approximately 2 hours prior to your study visit
- Apply the study sunscreen to the face (**only** for afternoon appointments)
- Afternoon Study visits**: 2 hours before your appointment** remove your facial makeup (do not need to remove your eye makeup). Apply the study facial cream approximately 2 hours prior to your study visit. **Do not** apply makeup or sunscreen to the face after use of the study cream.
- Please bring all study products to **all** your study visits

**Remember:**

- **Discontinue using** your current moisturizer, serum and/or any other skin care products
- **Do not change** your cleansing method, cleansing products during the study
- **Make note** of how your skin looks before and after each use of the study cream

Your feedback and honest opinions are important to us, and we appreciate your participation.

**The study product is for external use only.**

**Please keep all study products out of children’s reach.**

**VOLUNTEER INSTRUCTIONS**

**Prototype Applicator**

Pacific Bioscience Labs, Inc. would like to thank you for participating in this randomized, parallel arm home use test to evaluate signs associated with aging. You have been randomized to the prototype massage applicator group. The prototype massage applicator is designed to be used with the sonic handle. The purpose of this evaluation is to assess the effectiveness in treating signs associated with aging of a prototype massage applicator in applying a facial cream compared to manual application of a facial cream over an 8 week period.

You will be given a prototype massage applicator, a sonichandle, a charger, and a study facial cream to use at home twice per day (morning and bedtime) for the next 8 weeks.

After using the prototype massage applicator for the first time and at each study visit you will complete an online questionnaire. In order to adequately complete the study questionnaires, please pay close attention to the following:

- Appearance of overall facial fine lines/wrinkles as well as the fine lines and wrinkles on the forehead, cheek and upper lip/mouth
- Appearance of skin sagging on the face and neck areas
- Appearance of the lines between your eyes/eyebrows
- Overall appearance of the skin on your neck and neck folds
- Overall texture of the skin on your face and neck
- Characteristics of the anti-aging applicator head: Comfort, gliding effect, massaging effect, size, shape, vibration level

For the next 8 weeks you will be asked to cleanse your face twice per day (approximately the same time each morning and evening prior to bedtime) using the cleanser and cleansing method you normally use. After cleansing, rinse your face with warm water, pat dry with a towel, and use the prototype massage applicator to apply the study facial cream per instructions below.

**Remember: You are asked to discontinue using your current facial moisturizer, serum and/or any other facial product, but please do not change cleaning method, skin care cleansing products for the study duration.**

You will be instructed to:

- Discontinue using your current facial moisturizer, serum, and/or any other facial skin care products
- Use the study facial cream twice per day (morning and bedtime) on your face and neck area
- Apply the study sunscreen to the face and neck (morning only). Please avoid contact of the sunscreen with the eyes. In the event the sunscreen comes in contact with the eye, rinse the eye with water to remove.
- Closely examine your face and neck prior to and after using study facial cream

**Protocol for the day of each study visit:**

- Morning of visits: Cleanse your face using your normal cleanser and cleansing method (approximately same time each morning)
- Apply the study facial cream approximately 2 hours prior to your study visit
- Apply the study sunscreen to the face (**only** for afternoon appointments)
- Afternoon Study visits**: 2 hours before your appointment** remove your facial makeup (do not need to remove your eye makeup). Apply the study facial cream approximately 2 hours prior to your study visit. **Do not** apply makeup or sunscreen to the face after use of the study cream.
- Please bring all study products to **all** your study visits

**Remember:**

- **Discontinue using** your current moisturizer, serum and/or any other skin care products
- **Do not change** your cleansing method, cleansing products during the study
- **Make note** of how your skin looks before and after each use of the study cream

Your feedback and honest opinions are important to us, and we appreciate your participation.

**The study product is for external use only.**

**Please keep all study products out of children’s reach.**

Instructions for Use

- Use the prototype massage applicator to apply the study facial cream for **2 minutes 15 seconds twice per day** (morning and bedtime) on your face and neck area per instructions below:
- After cleansing your skin apply the recommended amount of the study facial cream to each of the following Treatment Zones:

| - **Zones** | - **Area** | - **Amount of study cream** |
| --- | --- | --- |
| - Zone 1 | - Forehead | - 1 scoop |
| - Zone 2 | - Cheeks and mouth | - 1 ½ scoops |
| - Zone 3 | - Neck and jawline | - 1 ½ scoops |

- Use the plastic spatula to scoop the recommended amount of the study facial cream for each identified Treatment Zones (Figure 1: amount of study cream per scoop)
- Evenly spread the study facial cream using your finger tips on Treatment Zones 1 and 2
- Use the device for the recommended amount of time
- Evenly spread the study facial cream using your finger tips on Treatment Zones 3
- Use the device for the recommended amount of time
- Set the electronic timer for the recommended time per Treatment Zone when using the prototype massage applicator

| **Zones** | **Area** | **Amount of time per Zone** |
| --- | --- | --- |
| Zone 1 | Forehead | 30 seconds |
| Zone 2 | Cheeks and mouth | 1 minute |
| Zone 3 | Neck and jawline | 45 seconds |
